# Supplementary material for: Experimental demonstration of separating the wave‒particle duality of a single photon with the quantum Cheshire cat
Source: Light Sci Appl. 2023 Jan 5;12:18. doi: 10.1038/s41377-022-01063-5 (PMC9813135; doi:10.1038/s41377-022-01063-5)
Supplement: Supplementary file 1 — Supplementary Material for: Experimental demonstration of separating the wave‒particle duality of a single photon with the quantum Cheshire cat [file 41377_2022_1063_MOESM1_ESM.pdf]

Supplementary Material for:

Experimental demonstration of separating the wave–particle duality of a single photon with the quantum Cheshire cat

Jia-Kun Li,[1,2,3] Kai Sun,[1,2,3*] Yan Wang,[1,2,3] Ze-Yan Hao,[1,2,3] Zheng-Hao Liu,[1,2,3] Jie Zhou,[4] Xing-Yan Fan,[4] Jing-Ling Chen,[4†] Jin-Shi Xu,[1,2,3‡] Chuan-Feng Li,[1,2,3§] and Guang-Can Guo[1,2,3]

[1]CAS Key Laboratory of Quantum Information, University of Science and Technology of China, Hefei 230026, China

[2]CAS Center for Excellence in Quantum Information and Quantum Physics,University of Science and Technology of China, Hefei 230026, China

[3]Hefei National Laboratory, University of Science and Technology of China, Hefei 230088, China

[4]Theoretical Physics Division, Chern Institute of Mathematics, Nankai University, Tianjin 300071, China

---

[*] ksun678@ustc.edu.cn
[†] chenjl@nankai.edu.cn
[‡] jsxu@ustc.edu.cn
[§] cfli@ustc.edu.cn

I. Details of the method used to extract the weak value

In the main text, we introduced the ITE theory, which links the slope of the linear fitting model and the weak values. Here, we provide an analytic proof of this method. Consider that observable $\hat{A}$ reads Hamiltonian $U(H,t)=e^{-Ht}$, where $H=\hat{A}$, when interaction time $t\rightarrow 0$, the detection probability can be written through Maclaurin's expansion of the first order:

$$N(U) = \left|\langle\psi_f|U|\psi_i\rangle\right|^2 = \left|\langle\psi_f|1-\hat{A}t+\cdots|\psi_i\rangle\right|^2$$
$$= N_0 - 2t\mathrm{Re}\langle\psi_i|\psi_f\rangle\langle\psi_f|\hat{A}|\psi_i\rangle + \cdots \quad (1)$$

Next, we divide the sides of the equation by N_0 and take the partial derivative to t :

$$\frac{\partial}{\partial t}\frac{N(U)}{N_0}\bigg|_{t\rightarrow 0} = -2\mathrm{Re}\frac{\langle\psi_f|\hat{A}|\psi_i\rangle}{\langle\psi_f|\psi_i\rangle} = -2\mathrm{Re}\langle\hat{A}\rangle_w \quad (2)$$

More specifically, in the experiment, to measure the weak value of the photons on the a side (right/left) with b properties (wave/particle), the non-unitary ITE operator is given as $U\left(\Pi_b^a,t\right)$ $=1-\Pi_b^a(1-e^{-t})$. The corresponding operation decreases the photon numbers in the path with ND filters whose transmission rate is $T=e^{-2t}$. Thus, the weak values should be written as follows:

$$\langle\Pi_b^a\rangle_w = -\frac{1}{2}\frac{\partial N}{\partial t}, \quad (3)$$

where $N=N(U)/N_0$. Therefore, after obtaining several data points and fitting the curve by a linear fitting model, minus half of the slope is the expected weak value.

II. Quantum state tomography results for the output states

As mentioned in the main text, here, we show all the state tomography results for output state $|\psi\rangle=\cos|H\rangle+\sin\alpha|V\rangle$ (the state at the outlet of the last BD in BS2) with different degrees of α in Figs S1–S5. For accuracy, the value of α is deduced from the tomography results instead of by directly reading the rotation angle of the HWP. When the measurement on one side is completed, tomography will be performed once again before switching to the other side, which ensures that the output state remains authentic. (In particular, for α = 0°/90°, the output state is simply a pure $|H\rangle/|V\rangle$ polarization state; therefore, there is no need to perform quantum state tomography after switching the measurement side, and both sides share the same tomography data). The actual value of **α** and the quantum tomography state results are shown in Table S1.

Table S1. Actual α and fidelity of the output state for each side when α varies from 0° to 90°

	Right		Left	
Expected α	Actual α	Fidelity	Actual α	Fidelity
0°	0°	99.79±0.13%	0°	99.79±0.13%
22.5°	21.00±0.51°	99.96±0.00%	20.99±0.58°	99.98±0.27%
45°	44.55±0.53°	98.26±0.37%	45.45±0.58°	98.05±0.35%
67.5°	66.85±0.46°	99.99±0.35%	66.83±0.46°	99.63±0.53%
90°	90°	99.90±0.07%	90°	99.90±0.07%

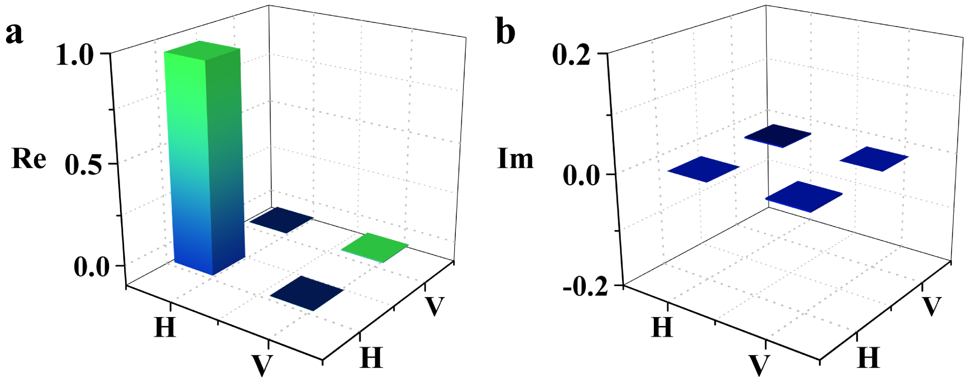

Fig. S1 Experimental quantum state tomography for output state $|\psi\rangle = \cos|H\rangle + \sin\alpha|V\rangle$ when α equals 0°. **a** Real and **b** imaginary parts of the reconstructed density matrix of the output state.

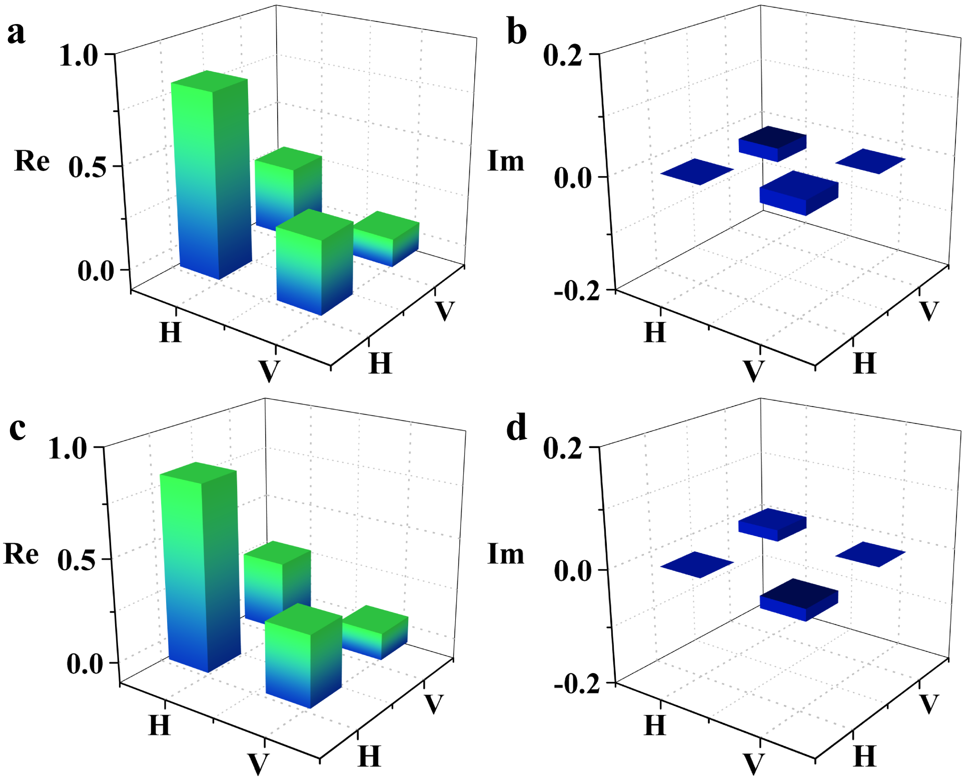

Fig. S2 Experimental quantum state tomography for output state $|\psi\rangle = \cos|H\rangle + \sin\alpha|V\rangle$ when α equals ~22.5°. **a** Real and **b** imaginary parts of the reconstructed density matrix of the output state for the right side (α = 21.00°). **c** Real and **d** imaginary parts of the reconstructed density matrix of the output state for the left side (α = 20.99°).

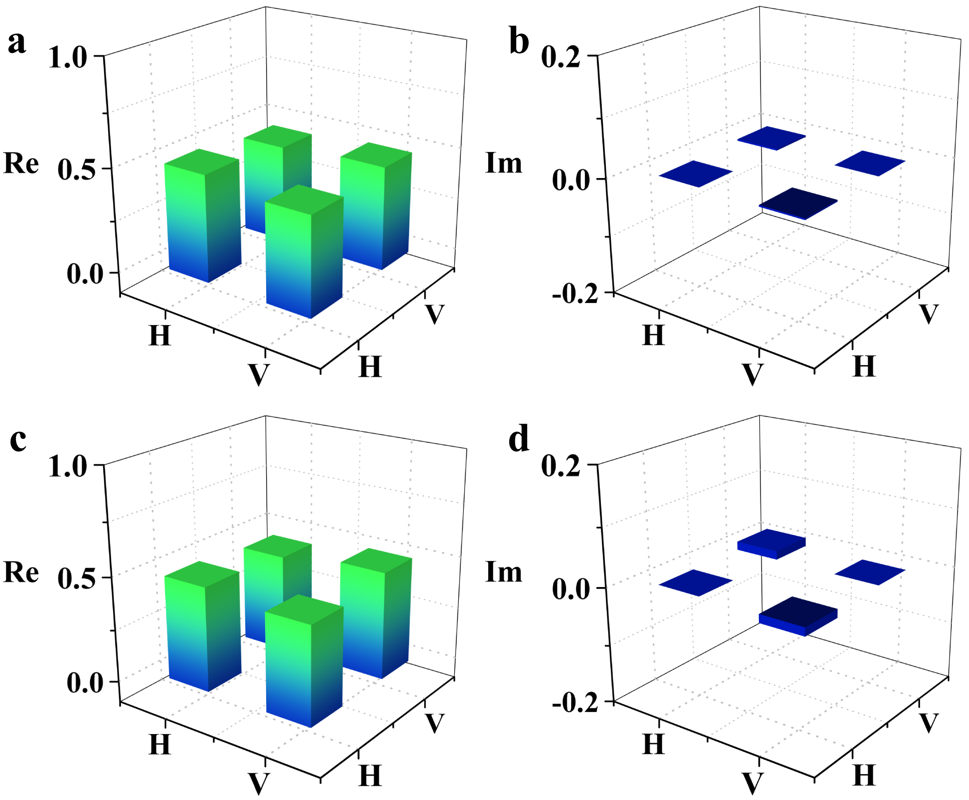

Fig. S3 Experimental quantum state tomography for output state $|\psi\rangle = \cos|H\rangle + \sin\alpha|V\rangle$ when α equals ~45°. **a** Real and **b** imaginary parts of the reconstructed density matrix of the output state for the right side (α = 44.55°). **c** Real and **d** imaginary parts of the reconstructed density matrix of the output state for the left side (α = 45.45°).

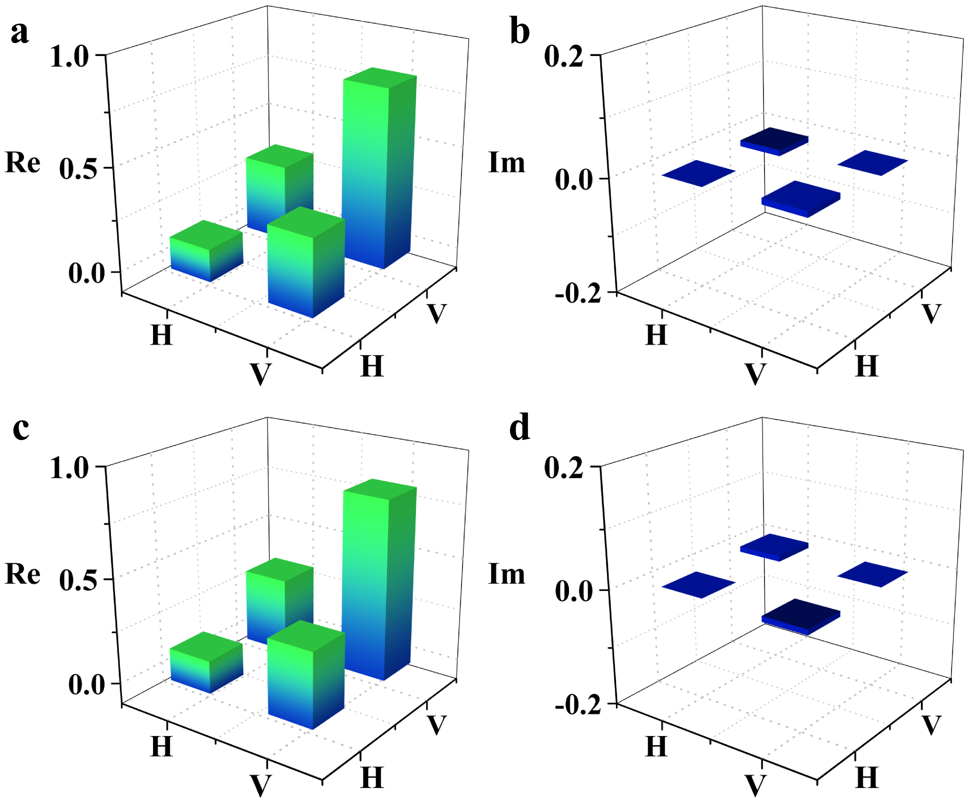

Fig. S4 Experimental quantum state tomography for output state $|\psi\rangle = \cos|H\rangle + \sin\alpha|V\rangle$ when α equals ~67.5°. **a** Real and **b** imaginary parts of the reconstructed density matrix of the output state for the right side (α = 66.85°). **c** Real and **d** imaginary parts of the reconstructed density matrix of the output state for the left side (α = 66.83°).

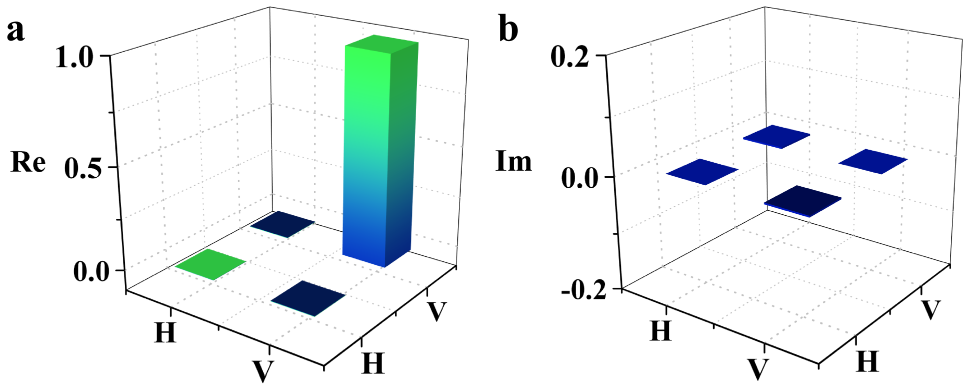

Fig. S5 Experimental quantum state tomography for output state $|\psi\rangle = \cos|H\rangle + \sin\alpha|V\rangle$ when α equals 90°. **a** Real and **b** imaginary parts of the reconstructed density matrix of the output state.

III. More experimental data for the linear fitting curves during the extraction of the weak values

The experimental data when inserting a series of ND filters with different transmission rates in the different paths in the interferometer for α ≈ 45° are shown in Fig. 3. Figs. S6–S9 present all other groups of data here. In particular, when α = 0° (90°), the light intensity in the down (up) path approaches zero. Thus, one cannot obtain the transmission rate for each point (namely abscissa *t*) regardless of what ND filter with a different transmission rate is put in the path. Thus, for these two specific situations, we cannot obtain the linear fitting curve but can deem the corresponding weak value to be 0 because the total coincidence counts remain almost unchanged in detector D1. In Table S2, we present the theoretically and experimentally obtained weak values for each side when α varies from 0° to 90°.

Table S2. Weak values for each path when α varies from 0° to 90°

$\langle \Pi_P^L \rangle_w$:

α	Slope of the linear fitting curve	Theoretical weak values	Experimental weak values
0°	0.006 ± 0.238	0	-0.003±0.119
20.99±0.58°	0.001±0.120	0	-0.001±0.060
45.45±0.58°	0.012±0.117	0	-0.006±0.059
66.83±0.46°	-0.025±0.096	0	0.013±0.048
90°	-	0	0

$\langle \Pi_P^R \rangle_w$:

α	Slope of the linear fitting curve	Theoretical weak values	Experimental weak values

0°	-1.905 ± 0.214	1	0.952±0.107
21.00±0.51°	-1.380±0.154	0.723	0.690±0.077
44.55±0.53°	-1.058±0.194	0.504	0.529±0.097
66.85±0.46°	-0.532±0.127	0.299	0.266±0.063
90°	-	0	0

$\langle \Pi_W^L \rangle_w$:

α	**Slope of the linear fitting curve**	**Theoretical weak values**	**Experimental weak values**
0°	-	0	0
20.99±0.58°	-0.618±0.118	0.277	0.309±0.059
45.45±0.58°	-1.131±0.126	0.504	0.565±0.063
66.83±0.46°	-1.322±0.135	0.700	0.661±0.067
90°	-1.959±0.246	1	0.980±0.123

$\langle \Pi_W^R \rangle_w$:

α	**Slope of the linear fitting curve**	**Theoretical weak values**	**Experimental weak values**
0°	-	0	0
21.00±0.51°	-0.058±0.169	0	0.029±0.085
44.55±0.53°	0.014±0.120	0	-0.007±0.060
66.85±0.46°	-0.056±0.136	0	0.028±0.068
90°	0.052±0.253	0	-0.026±0.127

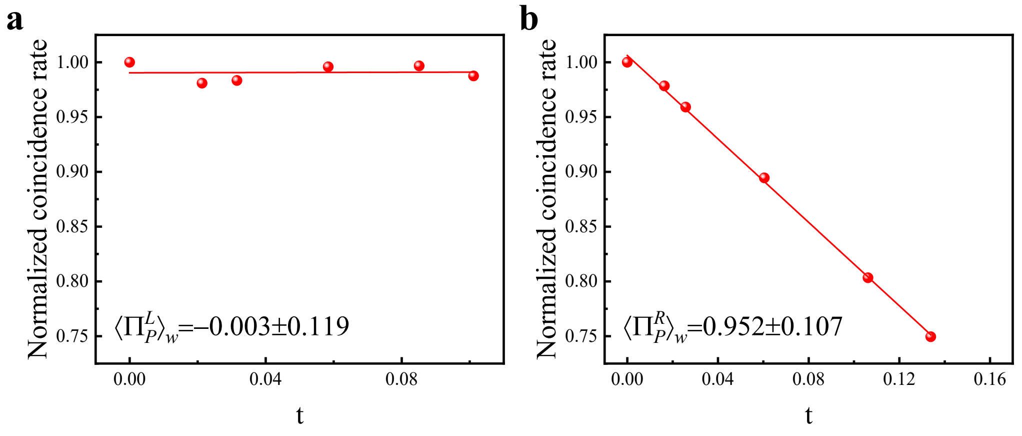

Fig. S6 Experimental data when inserting a series of ND filters with different transmission rates in the different paths of the interferometer for α = 0°. **a and b** Linear fitted line for $\langle \Pi_P^L \rangle_w$ and $\langle \Pi_P^R \rangle_w$, respectively. The corresponding weak value is displayed in the lower-left corner of each figure.

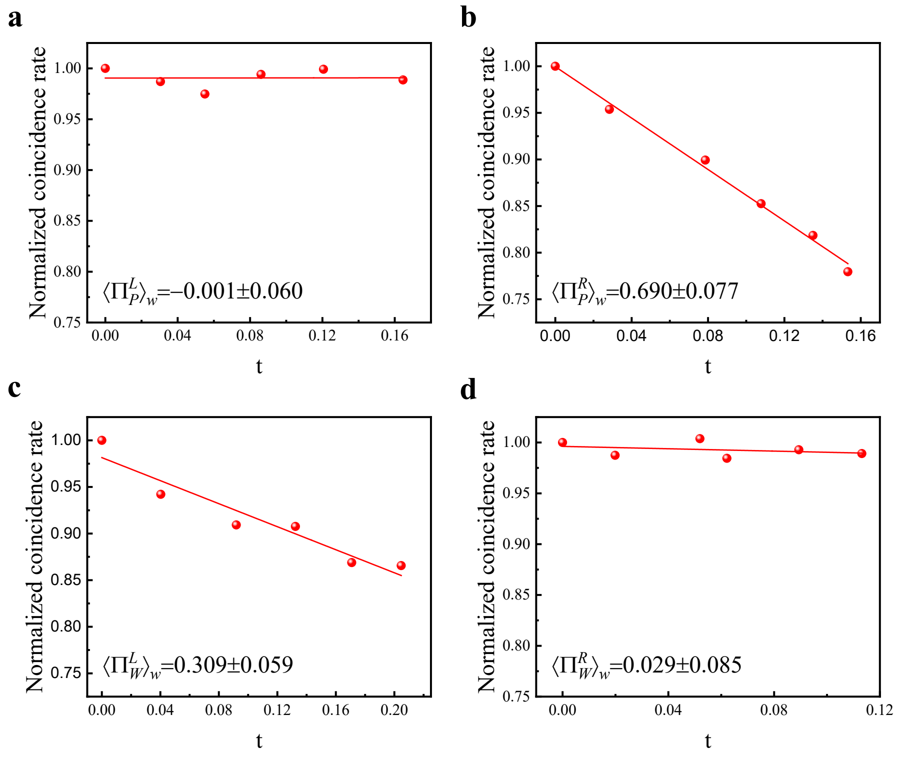

Fig. S7 Experimental data when inserting a series of ND filters with different transmission rates in the different paths of the interferometer for $\alpha \approx 22.5°$. **a–d** Linear fitted line for $\langle\Pi_P^L\rangle_w$, $\langle\Pi_P^R\rangle_w$, $\langle\Pi_W^L\rangle_w$, and $\langle\Pi_W^R\rangle_w$, respectively. The corresponding weak value is displayed in the lower-left corner of each figure.

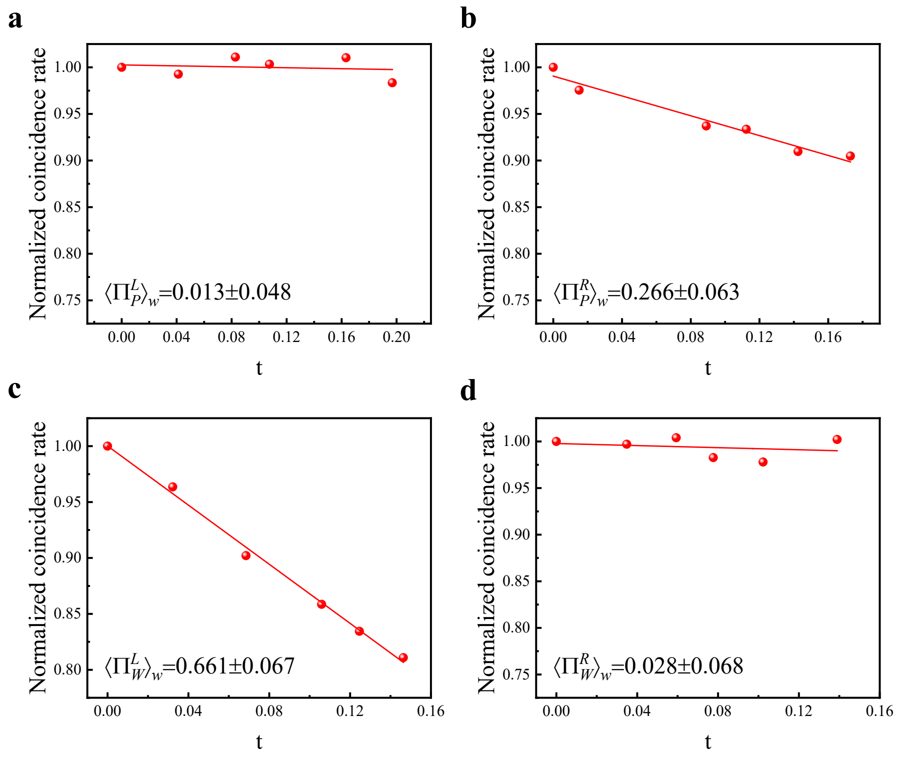

Fig. S8 Experimental data when inserting a series of ND filters with different transmission rates in the different paths of the interferometer for α ≈ 67.5°. **a–d** Linear fitted line for $\langle\Pi_P^L\rangle_w$, $\langle\Pi_P^R\rangle_w$, $\langle\Pi_W^L\rangle_w$, and $\langle\Pi_W^R\rangle_w$, respectively. The corresponding weak value is displayed in the lower-left corner of each figure.

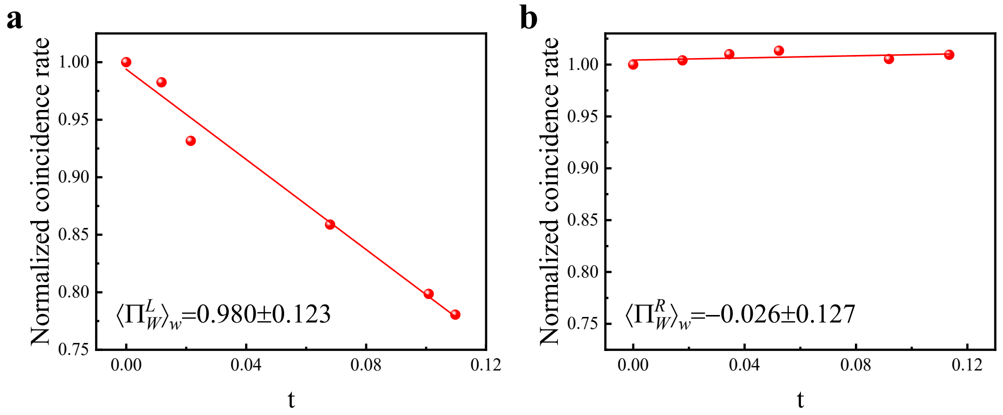

Fig. S9 Experimental data when inserting a series of ND filters with different transmission rates in the different paths of the interferometer for α = 90°. **a and b** Linear fitted line for $\langle\Pi_W^L\rangle_w$ and $\langle\Pi_W^R\rangle_w$, respectively. The corresponding weak value is displayed in the lower-left corner of each figure.
